# Evaluation of the leptin receptor in human spermatozoa

**DOI:** 10.1186/1477-7827-8-17

**Published:** 2010-02-23

**Authors:** Leila Hatami-Baroogh, Shahnaz Razavi, Hamid Zarkesh-Esfahani, Marziyeh Tavalaee, Somayeh Tanhaei, Kamran Ghaedi, Mohamad Reza Deemeh, Farzaneh Rabiee, Mohammad Hossein Nasr-Esfahani

**Affiliations:** 1 Department of Biology, Science and Culture University, Tehran, Iran; 2 Department of Reproduction and Development, Royan Institute for Animal Biotechnology, ACER, Isfahan, Iran; 3 Department of Anatomy, Isfahan Medical University, Isfahan, Iran; 4 Department of Biology, Faculty of Sciences, University of Isfahan, Isfahan, Iran; 5 Department of Cell and Molecular Biology, Royan Institute for Animal Biotechnology, ACECR, Isfahan, Iran; 6 Isfahan Fertility and Infertility Center, Isfahan, Iran

## Abstract

**Background:**

Leptin, a 167 amino acid peptide hormone, profoundly effects reproduction exerting its biological effects via interaction with the leptin receptor (ObR) which is widely expressed on peripheral tissues. In this study, we have attempted to assess leptin receptor expression in the spermatozoa of fertile males and those diagnosed with male factor infertility; both at the mRNA or protein levels.

**Methods:**

Semen samples were collected from fertile males and individuals with male factor infertility. In order to evaluate leptin receptor expression several techniques were utilized, including: reverse transcriptase-polymerase chain reaction (RT-PCR), immunostaining, flow cytometry, and western blotting. Mononuclear cells isolated from volunteers' peripheral blood were used as positive controls for leptin receptor expression.

**Results:**

leptin receptor was noted on mononuclear cells but we were unable to detect this receptor on spermatozoa at the protein level. Leptin receptor expression was detected on peripheral blood mononuclear cells (PBMCs) as positive controls; however it was not detectable on the spermatozoa of both groups by immunofluorescence microscopy or flow cytometry. Furthermore, positive expression of the ObR long isoform as assessed by RT-PCR was observed in the sperm of only four cases, whereas expression of beta-Actin, a house keeping gene, and HspA2, a testis specific gene, was present in all cases.

**Conclusion:**

The long isoform of leptin receptor may not be present on human sperm. Species difference may be accounted for diverse reproductive physiology which depends on metabolic requirement. Leptin receptor expression at the mRNA level in some individuals may be related to contamination by other cells in semen.

## Background

Hormones play a significant role in the unique and complex process of sperm production, many of which have been studied extensively. However, their precise role remains to be elucidated [1-3]. Leptin is a newly identified hormone with 167-amino acids produced by the obese gene [2] and its tertiary structure consists of four alpha helices connected by two long and one short loop [4]. This hormone resembles cytokines such as the granulocyte colony-stimulating factor (G-CSF) [5]. It is mainly secreted from adipocytes [2] and seminiferous tubules [6]. Leptin is involved in many biological processes, including: satiety, regulation of neuroendocrine systems, energy expenditure, haematopoiesis, angiogenesis, puberty and reproduction [7-9]. Leptin, as a nutritional signal, influences the secretion of luteinizing hormone (LH) by acting on the CNS as a neuroendocrine hormone [10].

Male mice lacking the leptin gene (ob/ob) are infertile and this effect is reversed by leptin treatment [8]. The role of leptin in controlling male reproductive function is controversial due to both stimulatory and inhibitory functions. It is believed that leptin regulates gonadal functions indirectly via the central neuroendocrine system and directly via the peripheral tissue membrane receptors [11]. Unlike leptin, its receptor has several isoforms; the full-length OB-Rb form [12] and several short forms that are generated by alternative splicing: OB-Ra, OB-Rc, OB-Rd, OB-Rf, and OB-Re, which lack signal transducing capabilities [13,14] and primarily differ in their cytoplasmic domain lengths [15-19]. The full-length OB-Rb is mainly expressed in the hypothalamus and expression of this receptor in testis appears to be species specific. In rodents, leptin receptor (ObR) mRNA is expressed in the Sertoli cells of adult rats [20] while leptin receptor immunoreactivity is confined to the Leydig cells of rats and the germ cells of mice [21,22] In addition, leptin receptor are present in human testicular tissue [14] and inhibit human chorionic gonadotropin (HCG)-stimulated testosterone secretion from rat Leydig cells in culture [23]. Leptin receptor has also been identified in boar [24] and pig spermatozoa [25]. In humans, the presence of leptin receptor has been reported in seminiferous tubules [26], however only Jope et al. (2003) have reported that seminal plasma and sperm contain this receptor [27]. Leptin receptor has been reported to be present in sperm of certain species but there are also reports claiming its absence in other species. In case of human, the reports are even more controversial. There are conflicting reports about presence of Leptin receptor in human sperm, therefore, the aim of this study was to evaluate presence of Leptin receptor in fertile and infertile males at both mRNA and protein levels. Our results demonstrated the absence of Leptin receptor at mRNA level in most of the cases and despite using several commercial and non-commercial antibodies and different techniques; we were unable to detect leptin receptor at protein level in spermatozoa of both groups.

## Methods

### Sample collection and preparation

Semen samples were collected from 50 individuals diagnosed with male factor infertility and 22 normal healthy males who were referred to the Isfahan Fertility and Infertility Center. Infertility is classically defined as a state in which a couple desiring a child, is unable to conceive following 12 months of unprotected intercourse. Subjects in the control group were healthy volunteers who had at least one child. All subjects signed an informed consent prior to participating in the study. The study was approved by the local ethics committee of Royan Institute and Isfahan fertility and Infertility Center.

Spermatozoa were diluted in PBS and isolated from semen samples by centrifugation (200 g for 10 minutes). The washing procedure ensured a reliable separation of spermatozoa from seminal plasma. Peripheral blood was collected into heparinized tubes and peripheral blood mononuclear cells (PBMCs) were isolated using a density gradient (Lymphoprep Nycomed, Norway) according to the manufacturer's recommendations. PBMCs were washed twice with PBS and used as positive controls for leptin receptor expression.

### RNA isolation and mRNA analysis

Total RNA was extracted from sperm or PBMCs with RNXplus (RNA isolation reagent, Cinagen, Iran). Total RNA (1 ug) was reverse-transcribed and first-strand complementary DNA (cDNA) was synthesized using commercial kits (Superscript II RT, Fermentase, Germany) according to the manufacturers' protocols. Polymerase chain reaction was performed with 2 microlitre of cDNA preparation, using specific primers that were previously published and checked on the basis of gene sequences identified with the BLAST search for Ob-Rb [28], HspA2 (a testis specific heat shock protein) and beta-Actin (housekeeping gene) [Table 1].

**Table 1:** Oligonucleotide sequences were used in RT-PCR analysis

Transcripts		Sequence direction (5'-3')
**Leptin-R**	Sense	GAA GAT GTT CCG AAC CCC AAG AAT G
	Antisense	CTA GAG AAG CAC TTG GTG ACT GAA C
**HspA2**	Sense	TTG TTG GAA GTC TTT GGT ATA
	Antisense	CAT TTG CAT TTA TGC ATT TGT
**β-Actin**	Sense	CGT GAC ATT AAG GAG AAG CTG TGC
	Antisense	CTC AGG AGG AGC AAT GAT CTT GAT

### Immunocytochemistry for leptin receptor detection

To assess leptin receptor expression in sperm and PBMCs, 10^6 ^cells were diluted in 200 μl of PBS containing 1% BSA and left for 30 minutes at room temperature. Then, cells were treated with a 1:1000 dilution of anti-human Leptin receptor antibodies using either N-20 goat anti-human OB-R antibody (Santa Cruz Biotechnology, Inc., Santa Cruz, CA), F-18 goat anti-human OB-R antibody (Santa Cruz Biotechnology, Inc., Santa Cruz, CA), or 9F8 mouse monoclonal anti-human OB-R antibody (gift from Professor Richard Ross, Sheffield University, UK) and incubated on ice for 2 hours. Samples were washed twice with PBS and incubated with the appropriate secondary antibody conjugated with FITC (Fluorescein isothiocyanate) and incubated on ice for 1 hour in the dark. Cells were washed twice, transferred to slides, and examined immediately with a fluorescence microscope (Olympus BX51, Japan).

### Flow cytometry assay

Sperm cells (5-10 million/ml) were incubated with 1% BSA in PBS for 30 minutes at room temperature and either 1 microlitre of primary antibody (9F8Ab, mouse monoclonal anti-human Leptin receptor antibody) or isotype-matched control antibody (Serotec, Oxford, UK) was added to the cells and incubated for 1 hour on ice. Cells were washed twice with PBS containing 1% BSA, then incubated with 1 microlitre of biotinylated goat anti-mouse IgG antibody (Calbiochem, Nottingham, UK). Cells were washed twice and incubated with 1 microlitre of Streptavidin-R-PE (Serotec, Oxford, UK) for 1 hour on ice in the dark. Finally, cells were washed twice before analyzing by flow cytometry on a FACS Calibur (Becton Dickinson, San Jose, CA, USA) flow cytometer.

### Western blot

Western blot analysis was carried out according to Nasrabadi and Henkel et al. [29,30]. Briefly, sperm pellets were treated with 10% (w/v) trichloroacetic acid in acetone with 0.07% (w/v) dithiothreitol (DTT) at -20°C for 1 hour. The suspension was centrifuged for 15 minutes at 16000 g. Pellets were washed with ice-cold acetone, incubated at -20°C for 30 minutes and centrifuged at 4°C at 12000 g for 15 minutes. The pellets were subsequently lyophilized. The sample powder was then solubilized in lysis buffer [9.5 M urea, 2% (w/v) CHAPS, 0.8% (w/v) Pharmalyte pH 3-10, 1% (w/v) DTT] and protein concentration was assessed by the Bradford assay (Bio-Rad) using BSA as the standard. The prepared samples were electrophoresed under reducing conditions on 10% SDS-PAGE gels (Bio-Rad, Munich, Germany) and then transferred to PVDF membranes (Bio-Rad, Munich, Germany). A page ruler prestained protein ladder (SM 0671; Fermentas, Germany) was applied for evaluation of molecular weights. Membranes were blocked overnight in 10% skim milk (Merck, Germany) in PBS and incubated with primary different antibodies against human Leptin receptor as mentioned above or anti-β-tubulin antibody, as a housekeeping gene or internal control (Sigma, USA) for 60 minutes, respectively. The blots were washed once for 15 minutes and then 3 times; each time for 5 minutes using a Tris buffer that contained 0.05% Tween 20 (Sigma, USA). For detection of primary antibodies, blots were incubated with the appropriate HRP (horseradish peroxidase) conjugated secondary antibodies for 1 hour. The washing procedure was repeated and bound secondary antibodies were detected using ECL advanced western blotting detection (GE Healthcare) and visualized by x-ray films (Agfa, Mortsel, Belgium). Recombinant human leptin R Fc Chimera (R&D Systems, USA) were used as positive controls.

## Results

Semen samples were collected from 50 individuals with only male factor infertility and 22 normal healthy males who were referred to the Isfahan Fertility and Infertility Center. In this study, out of 50 patients, 24%, 36% and 40% were grouped into oligoasthenoteratozoospermic, asthenoteratozoospermic, and teratozoospermia categories, according to WHO criteria, respectively [31]. Density ranges were from 5 to 80 million/ml, with a mean of 36.14 ± 25.80. The percentage of mean abnormal sperm morphology ranged from 73% to 96% (mean, 79.03 ± 9.29). Furthermore, the percentage of motility ranged from 00% to 95% with a mean of 66.17 ± 26.48.

**Figure 1:**
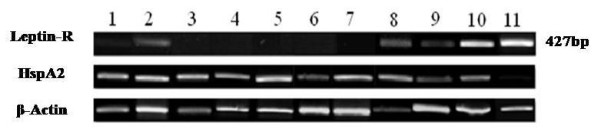
**RT-PCR results of Ob-Rb, HspA2 and β-Actin expression in sperm samples of four fertile (lanes 1-4) and six infertile individuals (lanes 5-10), and in PBMCs (lane 11)**.

**Figure 2:**
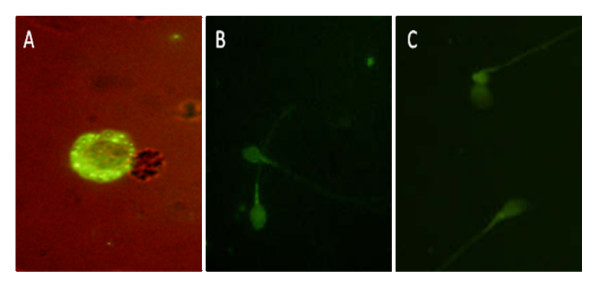
**Expression of ObR in PBMCs as positive control (A), in sperm of fertile (B), and infertile (C) individuals**. Scale bar 100 μm.

RT-PCR revealed the absence long isoform of Leptin receptor expression in 20 out of 23 infertile individuals and a weak expression in 1 out of 10 fertile individuals (Figure 1). In addition to RT-PCR, immunostaining was carried out for 50 infertile and 22 fertile individuals. Unlike PBMCs (positive control), spermatozoa from fertile and infertile individuals were negative for the leptin receptor extracellular domain after incubation with an anti-leptin-receptor antibody (Figure 2A, B, C).

**Figure 3:**
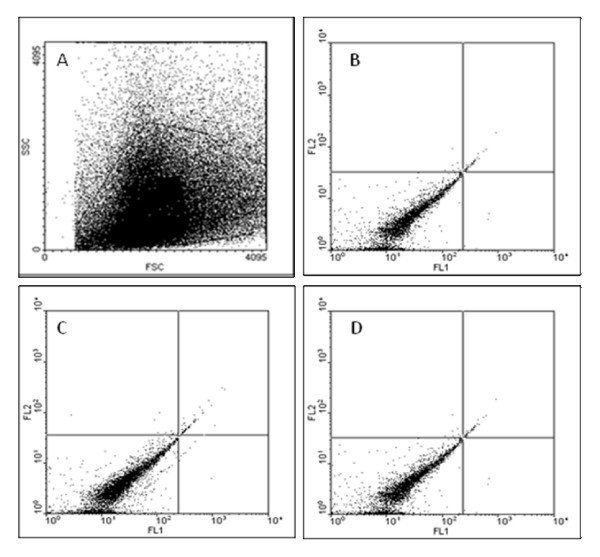
**Typical flow cytometry analyses of human spermatozoa**. ObR expression was not detectable in fertile and infertile individuals. Population of semen sample (A), Negative control (B), Isotype control (C), Treated with primary and secondary antibodies (D). There was no difference between the groups.

**Figure 4:**
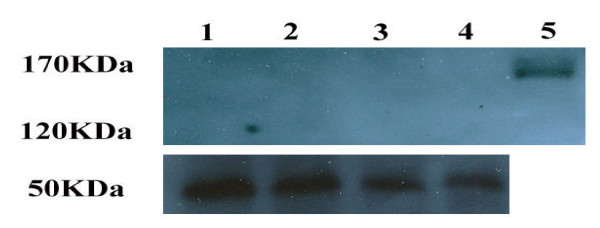
**Western blot analysis of ObR in sperm from two fertile (lanes 1 and 2) and two infertile individuals (lanes 3 and 4)**. Recombinant human leptin R Fc Chimera (lane 5). 170 and 120 KDa bands are defined as ObR while the 50 KDa band defines the house keeping gene beta-tubulin.

HspA2 expression, as a marker of testis specific marker and marker of sperm maturity, was also assessed. Figure 1 show that amplification of beta-Actin and HspA2 were present in all cases. Flow cytometry and western blot analysis were carried out on 10 fertile and 20 infertile individuals which also revealed the absence of Leptin receptor on the sperm of fertile and infertile individuals (Figures 3 and 4).

## Discussion

The hormonal link between fundamental function such as food intake and energy homeostasis with energy storage is believed to be mediated by leptin, and has been shown to play an important role in fertility in both male and female mice. These tasks are carried out by leptin-leptin receptor interactions in target tissues [7,8]. Leptin receptor (ObR) belongs to the family of cytokine receptors and considering the paramount role of leptin in physiology and reproduction, much interest has been focused on leptin receptor during the past few years[5].

Ishikawa has reported that in humans, leptin receptor have been shown to be present in testicular tissue and confined only to Leydig cells and are not expressed by Sertoli cells, germ cells and spermatozoa [32]. In addition, recent study by Li et al further confirmed the absence of mRNA of leptin receptor in human spermatozoa [33]. This observation further confirms the results of this study that leptin receptor is not expressed by spermatozoa both at mRNA and protein level, proved by both RT-PCR, immunostaing using different antibodies and by western blot analysis. In addition, these authors showed that the percentage of Leydig cells expressing leptin receptor increased in Sertoli only cell syndrome when compared to control individuals [32]. They did not observe such a difference between varicocele or with obstructive azoospermia with normal individuals. Accordingly, it was not unexpected to see the lack of leptin receptor expression in both fertile and infertile individuals in this study. Chen et al. (2009) also showed an increased testicular expression of leptin and its receptor in rats as an experimental varicocele model [34], they also conclude that leptin receptor was mainly observed in interstitium, confirming the pervious study of Iskikawa et al [32]. Therefore leptin may exert local effects on both the function of testis and spermatogenesis.

The results of this study showed that leptin receptor was detectable on the surface of human PBMCs as reported before [28], but were undetectable in sperm of most of subjects. A previous study has reported the presence of leptin receptor mainly on acrosome, the subequatorial area and the midpiece of boars [24]. In contrast to a report by Ishikawa et al. who did not histologically detect leptin receptor in germ cells and spermatozoa, Jope et al. and Li et al reported its presence on the tail region [27,33]. The difference in the leptin receptor location has been related to species differences. In pig, like boar, Leptin receptor has been detected in sperm head and it is believed that Leptin through autocrine short loop plays a physiological role in capacitation while in human it has been shown that Leptin has no significant role in capacitation and acrosome. Where the leptin to be present in the tail, one might envisage a role in sperm motility, which was not confirmed by Li et al [33]. This difference may account for species differences and for possible absence of Leptin receptor in sperm [25].

The leptin receptor (ObR) is a type I cytokine receptor family protein that has a significant amino acid sequence identity with gp130, GM-CSF receptor, and the LIF receptor. Therefore, since different polyclonal antibodies are used in these studies, the difference could be due to the lack of antibody specificity. Indeed, literature studies have revealed that when sperm are stained with antibodies against gp130 and GM-CSF, not only is the tail of the sperm stained, but the sizes of these molecules are within the leptin receptor range, which is 120-150 KD [35,36]. In addition, Jope et al. (2003) report that the detected leptin receptor is not of seminal plasma origin; however, they state that the molecular weight of the detected protein is of a similar molecular weight as the soluble seminal plasma form of the ObR [27]. In this study, the recombinant human leptin receptor was detected by western blotting, while the leptin receptor protein in the semen samples from fertile and infertile individuals was not detected (Figure 4). Additionally in this study, RT-PCR analysis showed a lack of leptin receptor expression in 29 out of 33 infertile and fertile individuals. In only four cases was the leptin receptor present, as indicated by RT-PCR analysis. One explanation for this observation could be the presence of other cell types in the semen, indeed, as shown in this study and other studies leukocytes express leptin receptor. These conclusions are in concordance with a previous study which could only detect leptin receptor in Leydig cells and not in mature spermatozoa in cross-sections of testis tissue [32,34]. Previous studies report an inverse correlation between leptin receptor expression by Leydig cells and the serum testosterone concentration in human testis, which suggested that over- expression of leptin receptor in Leydig cells leads to inhibition of testosterone production in infertile men [32]. Another possibility which could explain the absence of leptin receptor in human spermatozoa in this study and the studies by Ishikawa with Jope et al. or Li et al is that leptin receptor might reside on the acrosomal membrane and may be detectable before membrane modification which results from sperm capacitation and acrosome reaction [27,32,33]. This explanation appears to be invalid because if leptin receptor were present in the acrosomal membrane, it should have been detected by both mRNA and Western blot. In addition, leptin receptor has not been observed in any stages of spermatogenesis in the testicular sections. Therefore, considering the expression of leptin in germ cells and spermatocytes and the presence of leptin receptor only in human Leydig cells, the envisaged function for leptin and its receptor in testicular differentiation and germ cell proliferation [32] may appear to take place via testosterone production in Leydig cells which have the leptin receptor.

## Conclusion

The results of this study suggest that the long leptin receptor isoform is undetectable in human spermatozoa, however, further studies is required to resolve contradiction regarding presence, position and function of Leptin receptor in human sperm. Species differences have been suggested to be related on diverse reproductive physiology and it is depend on metabolic requirements.

## Competing interests

The authors declare that they have no competing interests.

## Authors' contributions

MHNE conceived and designed the study, interpreted the results performed the statistical analysis and drafted the manuscript. SHR Participated in designing the study. HZE carried out the flow cytometry and helped in drafting the manuscript. LHB and MT processed the samples and carried out the immunohistochemistry analysis. LHB, KGH, ST, and FR participated in RNA isolation, mRNA analysis and Western blot. MT interpreted the results, and commented on the draft manuscript. MRD processed the samples, and collaborated in collected samples and counseling the patient and obtaining the consent form. All authors have read and approved the final manuscript.
